# A high-quality chromosomal-level genome assembly of Greater Scaup (*Aythya marila*)

**DOI:** 10.1038/s41597-023-02142-x

**Published:** 2023-05-04

**Authors:** Shengyang Zhou, Tian Xia, Xiaodong Gao, Tianshu Lyu, Lidong Wang, Xibao Wang, Lupeng Shi, Yuehuan Dong, Honghai Zhang

**Affiliations:** College of Life Sciences, Qufu Normal University, Qufu, 273165 Shandong China

**Keywords:** Molecular ecology, Evolutionary genetics

## Abstract

*Aythya marila* is one of the few species of Anatidae, and the only *Aythya* to live in the circumpolar. However, there is a relative lack of research on genetics of this species. In this study, we reported and assembled the first high-quality chromosome-level genome assembly of *A. marila*. This genome was assembled using Nanopore long reads, and errors corrected using Illumina short reads, with a final genome size of 1.14 Gb, scaffold N50 of 85.44 Mb, and contig N50 of 32.46 Mb. 106 contigs were clustered and ordered onto 35 chromosomes based on Hi-C data, covering approximately 98.28% of the genome. BUSCO assessment showed that 97.0% of the highly conserved genes in aves_odb10 were present intact in the genome assembly. In addition, a total of 154.94 Mb of repetitive sequences were identified. 15,953 protein-coding genes were predicted in the genome, and 98.96% of genes were functionally annotated. This genome will be a valuable resource for future genetic diversity and genomics studies of *A. marila*.

## Background & Summary

Greater scaup (*Aythya marila*), the diving duck of the family Anatidae, which are distributed across Eurasia and North America. *A. marila* is one of the few Anatidae that breed in the Arctic, and the only duck belonging to the *Aythya. A. marila* which is a typical winter migrant. In winter, they migrate to the south to overwinter in shallow waters near the coast, estuaries, inland lakes, reservoirs, etc^1–3^. Little attention has been paid to this species because of extremely large range and population size. In fact, the population of *A. marila* has been declining in the past decades due to lack of effective conservation and reduced food quality^4,5^.

In recent years, with the development of high-throughput sequencing technology, genomes of more birds have been sequenced, providing a rich genetic resource for the study of species evolutionary mechanisms^6–10^. However, the published data does not include this species. Studies at the genetic level in this species were extremely rare due to lack of attention, and other few relevant studies were also related to diet, populations, and avian viruses^4,5,11^.

In this study, we present the first genome assembly of *A. marila* based on Nanopore sequencing and Hi-C sequencing technology. We used Nanopore sequencing data, Illumina sequencing data, and Hi-C sequencing data to assemble a genome (1,135.19 Mb) with scaffold N50 of 85.44 Mb, and 98.28% of the assembled bases were associated with the 35 chromosomes. This genome will be a valuable resource for many studies, including convergent evolution, population genomics, adaptive evolution, and comparative genomics, among others.

## Materials and methods

### Ethics statement

All animal experimental procedures were approved by the Biomedical Ethics Committee of Qufu Normal University (approval number: 2022001).

### Sampling and sequening

The experimental sample is a wounded male duck found during the wild bird survey in Jiangsu, China, which died unexpectedly during rescue. We dissected the sample and used the muscle for genome sequencing. Additionally, six transcriptomic samples (heart, kidney, liver, lung, pancreas, and spleen) from the same individual were collected and stored in an ultra-low temperature refrigerator at −80 °C, until samples were used for extracted genomic DNA and RNA.

Using Illumina® TruSeq® Nano DNA Library Prep kits to generate sequencing libraries of genomic DNA, we constructed a paired-end library with an insertion size of 350 bp, which was sequenced using Illumina HiSeq 4000 platform. Finally, a total of 60.77 Gb data (coverage of 45.30×) of 150 bp paired-end reads were generated. Simultaneously, using the PromethION platform with single-molecule real-time sequencing to sequence long reads. 122.55 Gb data were obtained, which covered 91.36 fold of the greater scaup’s genome. To assemble a chromosome-level genome of *A. marila*, a high-through chrome configuration capture (Hi-C) library was built and sequenced using Illumina PE150 platform. In total, we obtained 63.43 Gb raw reads. The six transcriptomic samples were used for RNA and sequenced using Illumina NovaSeq 6000 Platform, 43.9 Gb raw reads of all samples were obtained and used for subsequent gene prediction.

### Genome size estimation and assembly

To estimate the genome size, heterozygosity and the proportion of repetitive sequences in *A. marila*, we performed K-mer analysis using Illumina sequencing data by jellyfish (v2.2.7). While K = 17, the estimated genome size was 1,341.4 Mb, the heterozygosity was 0.47%, and the proportion of repetitive sequences was 42.28% (Table 1). Then, we used NextDenovo (v2.4.0) (https://github.com/Nextomics/NextDenovo) software to assemble the genome with Oxford nanopore technologies (ONT) long reads, and NextPolish^12^ (v1.3.1) was used to increase the precision of single base with Illumina short reads. We obtained a preliminarily assembly with size of 1,135.19 Mb after *de novo* assembly and base correction, which included 194 contigs, contig N50 was 34.42 Mb, and the proportion of GC was 41.94%.

**Table 1:** Genomic characteristics statistics while K = 17.

K-mer	Depth	Genome size (Mb)	Heterozygous rate (%)	Repeat rate (%)
17	31	1341.4	0.47	42.28

**Fig. 1:**
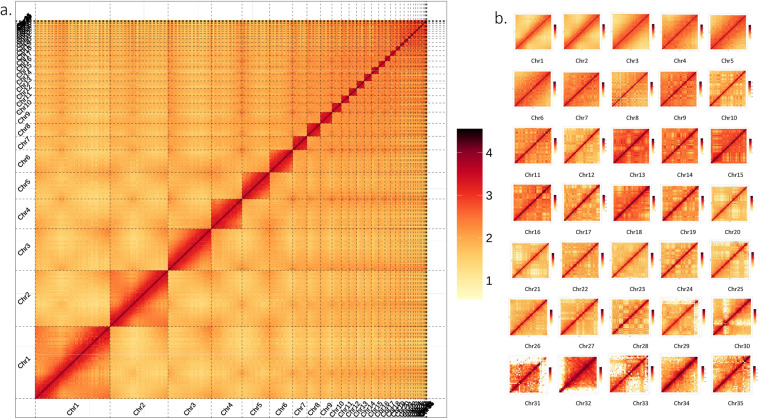
Visual heat map of the Greater Scaup’s Hi-C assembly. (**a**) The global heat map of all the chromosomes. (**b**) Heat maps of each chromosome.

ALLHiC^13^ (v0.9.8) and Juicebox (v1.11.08) software were used to mount the contigs in preliminarily assembly onto chromosomes. Contigs were sorted, pruned, and optimized based on the signal strength after Hi-C data were utilized to identify the interaction signals between Contigs. The interaction signals were then shown as a heat map using ALLHiC (Fig. 1). Using Juicebox to correct the position of contigs according to the intensity of chromosome interaction, and finally get a chromosome-level genome assembly with scaffold N50 of 85.44 Mb (Table 2). A total of 106 contigs were mapped to 35 chromosomes with lengths ranging from 2.11 Mb to 207.93 Mb, and 98.28% of the sequences were mapped to the chromosomes (Table 3).

**Table 2:** Assembly statistics of *A. marila*.

Sample ID	Contig length	Scaffold length	Number of Contig	Number of Scaffold
Total	1,135,187,360	1,135,194,460	196	125
Max	107,086,032	207,937,332	—	—
Number > = 2000	—	—	196	125
N50	32,460,729	85,439,399	11	4
N60	21,272,695	64,914,136	16	6
N70	18,686,645	32,655,158	21	9
N80	14,765,208	21,898,045	28	13
N90	7,683,303	13,253,193	39	19

**Table 3:** Summary of assembled 35 chromosomes of *A. marila*.

Sequeues ID	Cluster count	Sequeues Length	Sequeues ID	Cluster count	Sequeues Length
Chr1	6	207,937,332	Chr19	2	13,253,193
Chr2	9	160,322,976	Chr20	2	12,298,791
Chr3	10	120,566,604	Chr21	2	12,077,646
Chr4	9	85,439,399	Chr22	2	7,720,390
Chr5	2	77,230,333	Chr23	2	8,582,133
Chr6	2	64,914,136	Chr24	2	7,659,630
Chr7	3	39,865,393	Chr25	2	6,949,220
Chr8	6	38,335,560	Chr26	2	6,769,115
Chr9	2	32,655,158	Chr27	2	6,008,307
Chr10	2	26,959,460	Chr28	2	5,893,113
Chr11	2	22,384,702	Chr29	2	3,470,801
Chr12	2	22,081,037	Chr30	3	3,282,524
Chr13	2	21,898,045	Chr31	2	2,046,336
Chr14	2	21,377,020	Chr32	2	1,313,073
Chr15	2	20,214,777	Chr33	2	2,458,219
Chr16	2	18,084,087	Chr34	2	1,577,299
Chr17	2	16,432,712	Chr35	5	2,111,294
Chr18	3	15,468,941			
Placed	1,115,638,756 (98.28%)				
Unplaced	19,555,704 (1.72%)				
Total	1,135,194,460				

### Assessment of the genome assemblies

**Fig. 2:**
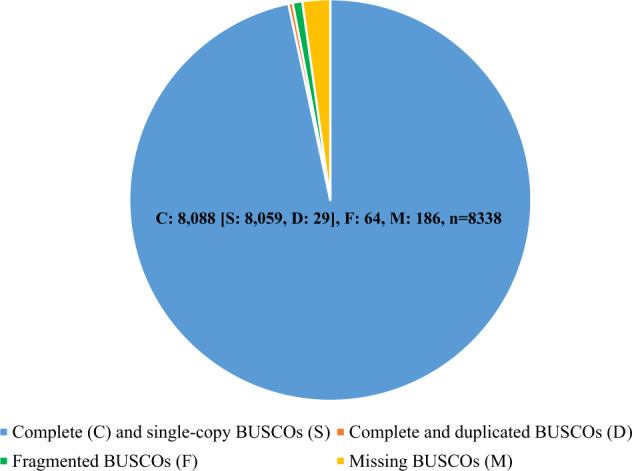
The BUSCO evaluation result of the Greater Scaup’s genome assembly.

To assess the accuracy of genome assembly, we used Burrows-Wheeler aligner^14^ (BWA) (v0.7.8) to map Illumina reads to the genome of *A. marila*. The reads matching rate was approximately 98.80%, indicating a good agreement between the reads and the assembled genome. Merqury^15^ (v1.3) was ran to evaluate assembly quality value (QV), and a high QV (42.14) indicates that this assembly is of good quality. Benchmarking Universal Single-Copy Orthologs^16^ (BUSCO) (v5.4.4) (use option “--augustus”) and Core Eukaryotic Genes Mapping Approach^17^ (CEGMA) (v2.5) were also used to assess the integrity of the assembled genome. The BUSCO results showed that 97.0% of the complete BUSCOs and 0.8% of the fragmented BUSCOs were found in 8338 single-copy orthologs of avers_odb10, and 2.2% of BUSCOs was missing (Fig. 2). Moreover, 238 of 248 core eukaryotic genes were detected using CEGMA. The above results indicate that the genome assembly of *A. marila* was complete and of high quality.

### Comparison with tufted duck genome assembly

**Fig. 3:**
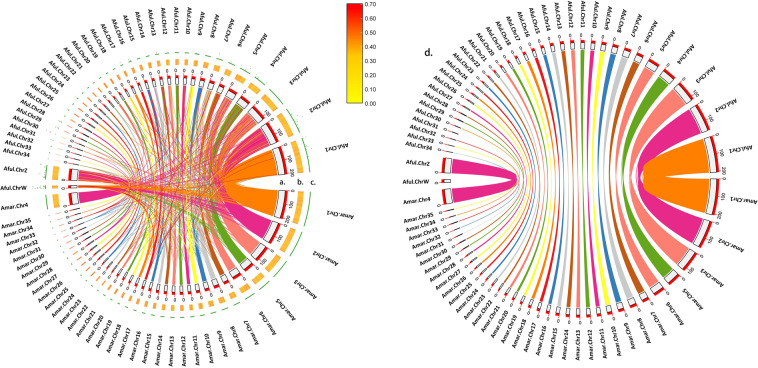
Chromosome-level synteny analysis of *A. marila* and *A. fuligula*, Amar means *A. marila*, Aful means *A. fuligula*. (**a**) Chromosomes length of *A. marila* and *A. fuligula* (Mb). (**b**) GC ratio (%) by 500 kb window. (**c**) Gap ratio (%) by 500 kb window. (**d**) The synteny plot of remove the non-orthologous links.

Mummer^18^ (v4.0.0) was used to identify the synteny between *A. marila* and tufted duck^19^ (*Aythya fuligula*) genomes to determine orthologous chromosome pairs, and we used TBtools^20^ (v1.112) to draw the synteny between their chromosomes. The synteny analysis shows that the chromosome sequences of the two species have good synteny, and each chromosome corresponds to multiple chromosomes of another species. There is one sex chromosome Z (Chr4) in *A. marila*, and it’s multiple autosome have some synteny with the sex chromosome W of *A. fuligula* (Fig. 3). Most of the chromosomes can correspond to the chromosome of the tufted duck, however both Chr31 and Chr32 shared synteny with chromosome 34 of the tufted duck, and the synteny was poor (Fig. 3d). Simultaneously, they also have synteny with some contigs. This may be due to the poor assembly quality caused by too small chromosomes size (Chr31, 2.05 Mb; Chr32, 1.03 Mb). In gap analysis, the chromosomes of *A. fuligula* genome assembly had a higher proportion of N bases (Fig. 3c). In general, the genome we assembled was of high quality.

### Annotation of repetitive sequences

We used a combined approach of *de novo* prediction and homology-based prediction to annotate repetitive sequences in the genome of *A. marila*. For *de novo* prediction, we used Tandem Repeats Finder^21^ (TRF) (v4.09) to detect tandem repeat sequences in the genome. RepeatModeler (v2.0.3), RepeatScout^22^ (v1.0.6) and RECON (v1.08) were used to build a database of transposable element (TE) family for *de nove* prediction. RepeatProteinMask and RepeatMasker (v4.1.2-p1) were used for homology prediction with Repbase database^23^ and Dfam database^24^, the species parameter used was chicken. Ultimately, we identified 154.94 Mb of repetitive sequences by all methods, accounting for 13.65% of the assembled genome (Table 4).

**Table 4:** Classification of repeat elements in *A. marila* genome.

	TRF		RepeatMasker		RepeatProteinMask		RepeatModeler	
	Length (bp)	% of Genome	Length (bp)	% of Genome	Length (bp)	% of Genome	Length (bp)	% of Genome
DNA	—	—	3,321,204	0.29	21,192	0.00	1,219,205	0.11
LINE	—	—	59,835,126	5.27	75,546,564	6.65	60,412,327	5.32
SINE	—	—	688,311	0.06	—	—	483,029	0.04
LTR	—	—	19,717,431	1.74	6,551,271	0.58	26,939,763	2.37
Unknown	—	—	495, 273	0.04	—	—	15,113,347	1.33
TRF	49,384,842	4.35	—	—	—	—	—	—
Total	49,384,842	4.35	84,057,345	7.40	82,119,027	7.23	104,167,671	9.18
	Non-redundancy		154,939,533 (13.65%)					

### Gene structure prediction

**Fig. 4:**
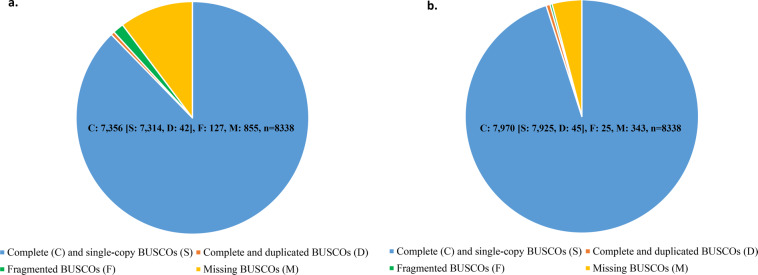
The evaluation result of BUSCO. (**a**) BUSCO result for 19,257 genes. (**b**) BUSCO result for 19,533 genes.

We used three methods to predict the protein-coding sequences in the genome of *A. marila*, *ab initio* prediction, homology-based prediction and RNA-Seq prediction. Three *ab initio* prediction software were used in the study, Augustus^25^ (v3.3.2), GlimmerHMM^26^ (v3.0.4), and Geneid^27^ (v1.4.5). For the six transcriptomic samples, the raw data were quality controlled and filtered out bad reads using fastp^28^ (v0.23.1) to obtain clean data. Then, the paired-end reads are assembled using SPAdes^29^ (v3.15.3). Next, the candidate coding regions in the transcript sequences are identified using TransDecoder^30^ (v5.5.0), and the sequences were clustered using CD-hit^31^ (v4.8.1) to remove redundant sequences. Eventually, the obtained protein sequences were used for subsequent prediction along with other downloaded sequences of related species. Genomes and annotation files of six related species^19,32–36^ (*Anser cygnoides, Anas platyrhynchos, Aythya fuligula, Cygnus olor, Cygnus atratus, Gallus gallus*) were downloaded from NCBI database to extract the longest transcript and translated them into protein sequences. Their protein sequences were matched to *A. marila* genome using Spaln^37^ (v2.4.6), and then the matched sequences were accurately spliced against the homologous protein-coding sequences using GeneWise^38^ (v2.4.1) software. Homology-based prediction results, RNA-Seq prediction results and *ab initio* prediction results are integrated using EvidenceModeler^39^ (EVM) (v1.1.1) to generate gene sets, and including masked repeats as input to the gene predictions. There were 19,257 genes after EVM integration. The BUSCO results showed that complete BUSCOs was 88.2%, fragmented BUSCOs was 1.5%, and missing BUSCOs was 10.3%, which was lower than the BUSCO results of genome (Fig. 4a). Thus, for some single-copy orthologs of aves_odb10, that ware complete in genome but missing or fragmented in predictive gene set, their annotation file were integrated into the gene set. Overall, a total of 19,533 protein-coding genes were predicted in *A. marila* genome, including 159 prematurely terminated genes and 273 partial genes. The average transcript length of this gene set was 23,126.79 bp, and the average coding sequence (CDS) length was 1,558.71 bp (Table 5). The BUSCO results showed 95.5% of the complete BUSCOs, 0.3% of the fragmented BUSCOs and 4.2% of the missing BUSCOs (Fig. 4b).

**Table 5:** The statistics of protein-coding genes annotated in *A. marila* genome.

	Gene set	number	Average transcript length (bp)	Average CDS length (bp)	Average exons per gene	Average exon length (bp)	Average intron length (bp)
*Ab initio*	Augustus	41,468	11,700.52	1,266.30	5.42	233.43	2,358.19
	Geneid	48,167	16,869.02	1,265.14	5.24	241.54	3,682.04
	GlimmerHMM	99,765	7,176.40	914.71	4.10	222.90	2,017.45
Homolog	*A. cygnoides*	35,485	19,574.11	1,179.17	6.82	172.87	3,159.92
	*A. platyrhynchos*	36,590	20,241.04	1,176.87	6.73	174.96	3,329.16
	*A. fuligula*	31,903	22,421.14	1,271.42	7.33	173.38	3,339.50
	*C. olor*	35,258	20,132.93	1,173.16	6.76	173.66	3,294.29
	*C. atratus*	31,863	21,669.31	1,264.08	7.31	172.87	3,232.51
	*G. gallu*	33,839	21,287.57	1,248.52	7.07	176.64	3,302.30
RNAseq	Heart	62,460	10,409.30	616.96	3.62	170.43	3,737.54
	Kidney	61,707	10,532.81	636.23	3.73	170.76	3,630.58
	Liver	54,111	10,053.47	636.21	3.80	167.45	3,364.06
	Lung	89,887	9,141.47	525.78	3.04	173.20	4,232.41
	Pancreas	48,767	10,065.86	655.17	3.95	165.90	3,190.99
	Spleen	72,901	10,079.22	587.42	3.37	174.14	3,999.53
EVM		19,257	23,878.78	1,569.93	8.97	174.93	2,797.50
Final set		19,533	23,126.79	1,558.71	8.91	174.91	2726.23

### Gene function annotation

Next, function annotation of predicted genes for *A. marila* using diamond^40^ against SwissProt^41^, TrEMBL^42^, Non-Redundant Protein Sequence Database (NR), Gene Ontology^42^ (GO), and Kyoto Encyclopedia of Genes and Genomes Orthology^43^ (KO) databases with e-value cutoff of 1e-5. The InterPro database^44^ is also used for function annotation by classifying proteins into families and predicting domains and important sites with Interproscan^45^ (v5.53). Finally, 15,789 genes were annotated, and accounted for 81.50% of all predicted genes of *A. marila* (Table 6).

**Table 6:** Annotation of predicted genes.

	Number	Percent (%)
Total	19,374	—
SwissProt	14,634	75.53
TrEMBL	15,568	80.35
InterPro	14,852	76.66
NR	15,751	81.30
KO	11,728	60.53
GO	11,761	60.71
Annotated	15,789	81.50
Unannotated	3,585	18.50

### Filtering and verification of gene set

**Fig. 5:**
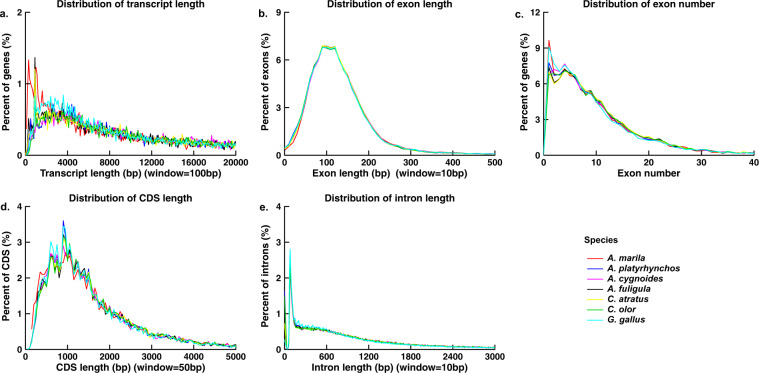
Comparison graph of the prediction gene in *A. marila* and related species. (**a**) Gene length distribution and comparison with other species. (**b**) Exon length distribution and comparison with other species. (**c**) Exon number distribution and comparison with other species. (**d**) CDS length distribution and comparison with other species. (**e**) Intron length distribution and comparison with other species.

We filtered the predicted gene set, because the number of predicted genes was more than in the six related species, and more than 3,000 genes could not be function annotated. We used OrthoFinder to find the orthologs of *A. marila* and six related species. There were 4,086 unassigned genes, and 3,421 of them were not annotated in any database (Table 7). Most of these genes (3,417/3,421) were predicted using at least one of the *de novo* prediction software, and only four genes are supported by other evidence. The BUSCO test was performed after removed unassigned genes without function annotations, the results showed no effect compared with before removed (Table 8; Fig. 4b). These suggests that these genes may only have specific sequences and patterns, rather than real genes^46^. After filtering the unassigned genes without annotation and 159 prematurely terminated genes, 15,953 genes remain, including 182 partial genes, and 98.96% of them were annotated. The filtered gene set of *A. marila* was compared with the longest transcript set of related species, it has similar structure (Table 9 and Fig. 5). Except *G. gallu*, the average transcript length, average CDS length, average exons number, and average intron length of *A. marila* were smaller than those of others. Simultaneously, the average exon length of *A. Marila* was the longest among all species. However, these differences were not significant. This may be due to *A. Marila* has a higher proportion of short genes (Table 5a).

**Table 7:** Orthofinder Statistics Result of PerSpecies.

	*A. marila*	*A. platyrhynchos*	*A. cygnoides*	*A. fuligula*	*C. atratus*	*C. olor*	*G. gallu*
Number of genes	19,374	16,649	15,752	15,371	15,330	15,858	17,923
Number of genes in orthogroups	15,288	16,459	15,399	15,323	15,248	15,627	17,085
Number of unassigned genes	4,086	190	353	48	82	231	838
Percentage of genes in orthogroups	78.9%	98.9%	97.8%	99.7%	99.5%	98.5%	95.3%
Percentage of unassigned genes	21.1%	1.1%	2.2%	0.3%	0.5%	1.5%	4.7%

**Table 8:** Busco results of the filtered gene set.

Type	Number
Complete BUSCOs (C)	7,970 (95.5%)
Complete and single-copy BUSCOs (S)	7,905 (95.0%)
Complete and duplicated BUSCOs (D)	45 (0.5%)
Fragmented BUSCOs (F)	25 (0.3%)
Missing BUSCOs (M)	343 (4.2%)
Total BUSCO groups searched	8338

**Table 9:** The comparison of the genetic structure from *A. marila* genome and related species.

Species	Gene number	Average transcript length (bp)	Average CDS length (bp)	Average exons number	Average exon length (bp)	Average intron length (bp)
*A. marila*	15,953	27,243.49	1,793.43	10.40	172.46	2,707.71
*A. cygnoides*	15,921	29,461.10	1,807.73	10.68	169.21	2,855.76
*A. platyrhynchos*	16,823	29,072.55	1,798.56	10.74	167.41	2,799.16
*A. fuligula*	15,565	29,899.77	1,815.99	10.92	166.35	2,831.99
*C. olor*	16,137	30,271.50	1,830.15	10.90	167.93	2,873.43
*C. atratus*	15,552	30,161.93	1,828.44	10.97	166.74	2,842.98
*G. gallu*	18,024	25,524.58	1,746.77	10.19	171.45	2,587.85

## Data Records

### All sequencing data had been uploaded to NCBI database

The genomic Illumina sequencing data, Nanopore sequencing data and Hi-C sequencing data were deposited at the Sequence Read Archive database of NCBI (accession numbers: SRR21672225^47^, SRR21672223^48^, and SRR21672224^49^).

The transcriptome data of 6 samples were deposited at the Sequence Read Archive database of NCBI (accession numbers: SRR21700073 - SRR21700078^50–55^).

The genome assembly of *A. marila* were deposited at GenBank database under the accession JAOQIG000000000^56^.

The annotation results of repeats sequences, gene structure and functional prediction were deposited at the Figshare database^57^.

## Technical Validation

Quality control was performed on Illumina, Nanopore, Hi-C, and RNA sequencing data. The Q20 of Illumina sequencing data were greater than 96% and Q30 were greater than 90%. The Q7 of Nanopore sequencing data were greater than 92%. The Q20 of Hi-C sequencing data were greater than 94% and Q30 were greater than 96%. And the Q20 of RNA sequencing data were greater than 97%, and Q30 were greater than 93%. These evidences suggest that the data are reliable and can be used in subsequent analyses (Tables 10, 11).

**Table 10:** Statistical information of Illumina, Hi-C and RNA sequencing data.

	Sample name	Raw Base(G)	Effective Rate(%)	Error Rate(%)	Q20(%)	Q30(%)
Illumina	FDSW210006884-4r_L1	58.84	99.72	0.04	96.56	91.7
	FDSW210006884-4r_L2	1.94	99.81	0.04	96.02	90.91
Hi-C	BDHC210000461-1A_L1	18.44	99.69	0.06	94.15	86.68
	BDHC210000461-1A_L2	7.11	99.77	0.04	95.7	89.67
	BDHC210000461-1A_L3	97.49	99.71	0.06	94.37	87.12
RNA-seq	BB_heart	9.38	96.49	0.03	97.44	93.4
	BB_spleen	6.58	93.04	0.03	97.45	93.24
	BB_liver	7.09	94.08	0.03	97.56	93.27
	BB_pancreas	7.2	92.58	0.03	98.18	94.65
	BB_lung	7.21	92.74	0.03	97.73	93.67
	BB_kidney	6.44	93.91	0.03	97.87	94.04

**Table 11:** Statistical information of Nanopore sequencing data.

Read type	Read base (Gb)	Read length (N50) (bp)	Read quality (mean)	Read quality >Q7 (%)
Nanopore reads	122.56	24,287	12.6	92.50%

## Data Availability

No special codes or scripts were used in this work, all data processing commands and parameters were executed according to the manuals and protocols of the corresponding bioinformatics software.
